# Supplementary material for: Molecular Docking of Natural Compounds for Potential Inhibition of AhR
Source: Foods. 2023 May 11;12(10):1953. doi: 10.3390/foods12101953 (PMC10217167; doi:10.3390/foods12101953)
Supplement: Supplementary file 1 [file foods-12-01953-s001.zip › foods-2363279-supplementary.pdf]

Molecular Docking of Natural Compounds for Potential Inhibition of AhR

Deborah Giordano, Angelo Facchiano, Stefania Moccia, Anna Maria Iole Meola, Gian Luigi Russo and Carmela Spagnuolo *

National Research Council, Institute of Food Sciences, 83100 Avellino, Italy; deborah.giordano@isa.cnr.it (D.G.); angelo.facchiano@isa.cnr.it (A.F.); stefania.moccia@isa.cnr.it (S.M.); iolemeola@hotmail.com (A.M.I.M.); glrusso@isa.cnr.it (G.L.R.);
* Correspondence: carmela.spagnuolo@isa.cnr.it

Supplementary materials

A

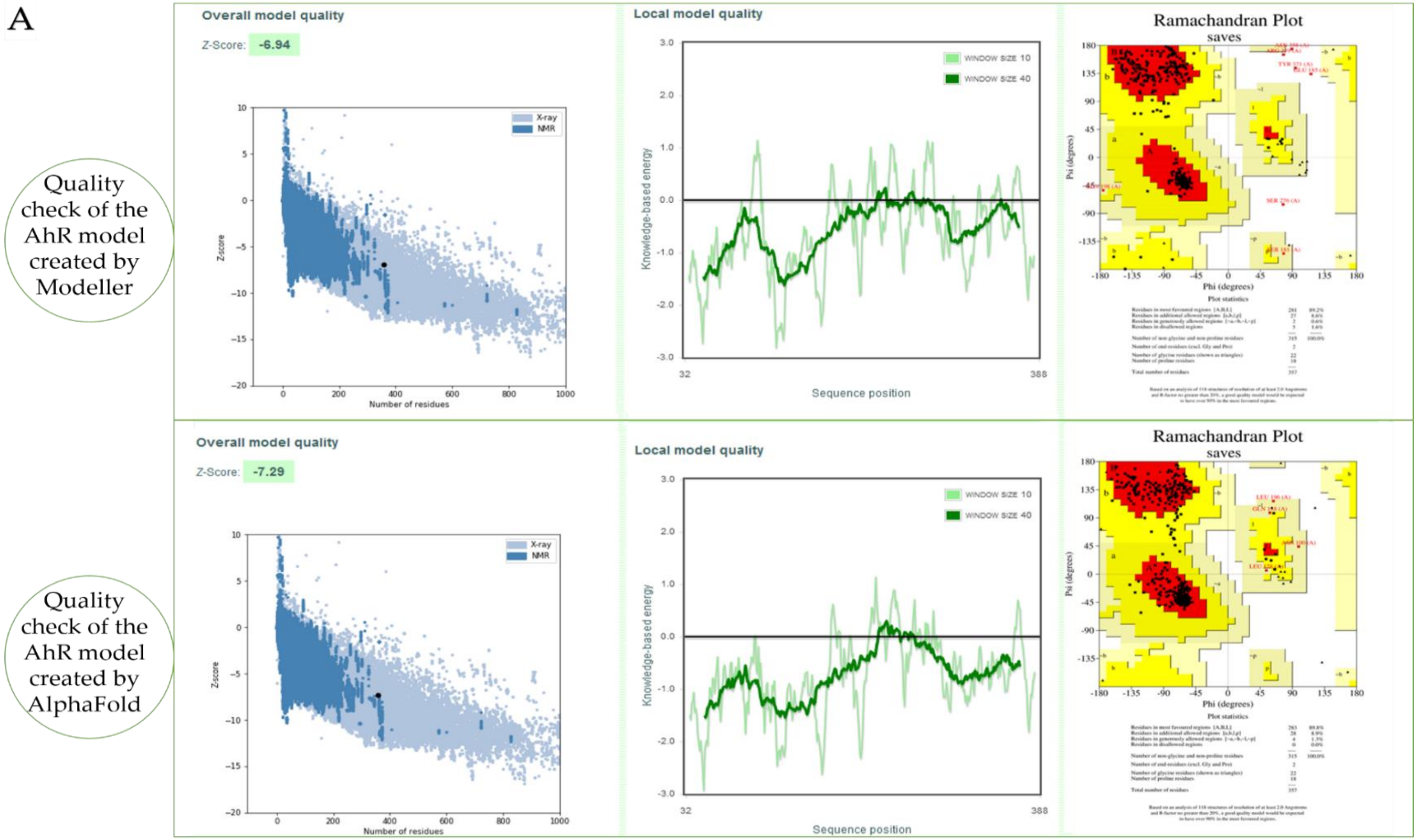

B

MODEL	Q-MEAN	Z-SCORE	RAMACHANDRAN PLOT REGIONS (most favoured - additional allowed - generously allowed - disallowed)
AhR	0.650	-6.94	89.2% - 8.6% - 0.6% - 1.6%
AhR PAS A	0.666	-5.51	89.9% - 8.4% - 0.8%- 0.8%
PAS B (mod1)	0.614	-2.89	86.8% - 9.2% - 0.0% - 3.9%
PAS B (MODEL 2)	0.551	-4.64	92.6% - 5.3% - 2.1% - 0.0%
AhR whole by AlphaFold	0.467	-6.14	73.2% - 17.7% - 3.9% - 5.2%
AhR by Alpha Fold	0.706	-7.29	89.8% - 8.9% - 1.3% - 0%.
AhR by Alpha Fold (PAS A)	0.667	-5.81	87.3% - 11.0% - 1.7% - 0.0%
AhR by Alpha Fold (PAS B)	0.806	-3.44	97.4% - 2.6% - 0.0% - 0.0%

Supplementary Figure S1. Quality of structural models of AhR: (A) Comparison of the Z-score, the local model quality, and the Ramachandran plot values between the AhR multidomain model generated by Modeller (upper side) and by Alpha Fold (lower side); (B) Table of the quality parameters for AhR domain and multidomain models generated in our study and for AlphaFold AhR model.

Supplementary Tables S1-S14. Detailed docking results related to the 14 hypothetical ligands tested: main docking results related to β-carotene (S1), lycopene (S2), xanthohumol (S3), hesperetin (S4), naringenin (S5), curcumin (S6), quercetin (S7), resveratrol (S8), cyaniding-3- glucoside (S9), epigallocatechin-3-gallate (S10), genistein (S11), hydroxytyrosol (S12), ellagic acid (S13), sulforaphane (S14). Residues underlined in the interacting residues column are involved in H-bonds formation.

Supplementary Table S1

DOCKING TYPE	LIGAND	RUN	ENERGY (Kcal/mol)	Ki	N° IN CLUSTER	POCKET	INTERACTING RESIDUES
BLIND	**β-CAROTENE**	5	-10.65(LBE=-12.57)	609.52 pM	32	D with different orientations	PHE82-PHE83-GLY96-GLY97-GLN98-VAL126-VAL128-PHE136-TYR137-CYS219-GLN240-GLY241-PHE266-ALA267-ASP329-TYR332-ILE349-VAL350
		71	-8.88(LBE=-9.99)	47.47 nM	13		ASP166-GLU169-GLN173-LEU178-PRO214-LEU215-MET216-GLU217-ARG218-CYS219-PHE220-ILE221-ARG223-RG339-LYS342-THR343
		49	-7.69(LBE=-8.29)	833.97 nM	12		ARG94-ASP99-GLY115-LEU119-GLN240-ILE268-ASP329-TYR332-ILE349-VAL350-ARG352-TRP362
FOCUSED		59	-7.13(LBE=-8.08)	1.19 μM	38	B	GLY344-GLU345-ASN366-ARG368-LEU369-TYR371-ASN373-GLY374-ILE380-THR382-ARG384
		85	-6.77(LBE=-7.64)	2.52 μM	16	Behind B (between PAS A and B)	ALA328-ASP329-TYR332-SER336-ARG339-THR343-GLU345-SER346-GLY347-ILE349-VAL350-TRP362-
		99	-6.64(LBE=-7.46)	3.43 μM	12		ARG339-GLU345-SER346-GLY347-ILE349-VAL350-TRP362-GLN364-ASP388-GLY391-THR392-HIS394
		3	-6.69(LBE=-7.01)	7.26 μM	12	Behind B	TYR332-GLU335-SER336-ARG339-ILE349-VAL350-ASP388-GLU389-GLY391-THR392-HIS394

Supplementary Table S2

DOCKING TYPE	LIGAND	RUN	ENERGY (Kcal/mol)	Ki	N° IN CLUSTER	POCKET	INTERACTING RESIDUES
BLIND	**LYCOPENE**	88	-8.24 (LBE=-9.96)	49.75 nM	4	Between A and B	ASP144-ARG223-LEU228-ASN230-SER231-PHE234-LEU235-ALA236-GLN273-PRO274-ILE280-ILE341-THR343-GLY344-GLU345-LEU369-TYR371-LYS372-PRO376
		82	-7.84 (LBE=-9.57)	96.83 nM	12	D	PHE82-PHE83-GLY96-GLY97-GLN98-VAL126-VAL128-PHE136-TYR137-GLN240-PHE266-ALA328-TYR332-ILE349-VAL350
		50	-7.08 (LBE=-7.57)	2.83 μM	8 (+6Run1)	Behind B (between PAS A and B)	GLU165-ASP166-GLN173-LEU178-ASN212-SER213-LEU215-MET216-GLU217-ARG218-CYS219-PHE220-ILE221-ARG223-ARG339-LYS342-THR343
		25	-6.59 (LBE=-7.10)	6.25 μM	8 (+7Run98)	D	TRP136-ALA177-LEU178-LEU215-MET216-GLU217-ARG218-CYS219-ILE349-VAL350-TRP362-LEU386-THR387
FOCUSED		92	-7.58	2.77 μM	1	PAS B (Down)	LEU293-PHE295-TYR322-GLN323-MET330-ALA334-HIS337-ILE341-LEU354-TYR371-PRO376-TYR378
		41	-5.40 (LBE=-6.53)	16.42 μM	9	Behind B (between PAS A and B)	TYR332-GLU335-SER336-ARG339-LYS342-ILE349-VAL350-TRP362-ASP388-GLY391-THR392
		67	-5.16 (LBE=-6.45)	18.82 μM	14 (+15Run20+4Run87 +6Run90)		THR343-GLU345-GLY347-ILE349-VAL350-TRP362-GLN364-TYR371-GLY391-HIS394
		91	-5.29 (LBE=-6.18)	29.51 μM	17 (+4Run85+10Run89)	B	ILE286-GLY344-GLU345-ASN366-ALA367-ARG368-LEU369-TYR371-LYS372-ASN373-GLY374-ILE380-THR382-ARG384

Supplementary Table S3

DOCKING TYPE	LIGAND	RUN	ENERGY (Kcal/mol)	Ki	N° IN CLUSTER	POCKET	INTERACTING RESIDUES
BLIND	**XANTHOHUMOL**	4	-9.68	79.94 nM	1	PAS A	THR131-TYR159-HIS175-PRO180-CYS183-THR184-THR194-GLY195-PRO197-PRO260-GLN262-LEU263-GLN171
		47	-9.05	212.48 nM	2	Between A and B	ASN123-TYR145-LEU228-ASN230-LEU235-PRO271-GLN273-LEU278-ILE280-THR382
		37	-8.32	791.60 nM	1	A (upper side)	LEU119-GLN120-LEU122-ASN123-ASP144-TYR145-CYS226-LEU228-LEU235-GLN273-ILE280-ARG281
		89	-8.04	1.27 µM	1	Between A and B	ASN123-TYR145-LEU235-PRO271-GLN273-PRO274-PRO275-LEU278-ILE280-ARG281-THR282-THR382
		84	-7.67	2.25 µM	2	PAS A	VAL200-VAL201-CYS202-TYR203-GLN207-SER213-MET216-LYS244-TYR245-HIS247
		97	-7.59	2.28 µM	5	Between A and B	GLN120-ASN123-TYR145-GLN273-PRO275-LEU278-ILE280-ILE286-ARG384
		14	-6.68	5.78 µM	7	D (upper side)	ASN95-GLY96-GLY97-GLN98-VAL128-TYR137-GLY241-LYS242-LYS244-PHE266
		6	-6.88	6.19 µM	6	PAS A	PHE82-SER90-GLN207-PRO210-SER213-LEU215-LYS242-LYS244-TYR245
FOCUSED		2	-6.80	10.44 µM	1	C	PHE287-THR289-HIS291-PHE295-PRO297-LEU308-LEU315-GLY321-PHE324-ILE325-HIS337-PHE351-LEU353-VAL363-TYR378-VAL381-GLN383
		80	-5.66	27.04 µM	16	B	TYR343-GLY344-ASN366-ALA367-LEU369-LEU370-TYR371-ILE380-VAL381-THR382
		57	-5.56	37.73 µM	14	B	THR343-GLY344-GLU345-ASN366-ALA367-ARG368-LEU369-TYR371-ILE380-VAL381-THR382

Supplementary Table S4

DOCKING TYPE	LIGAND	RUN	ENERGY (Kcal/mol)	Ki	N° IN CLUSTER	POCKET	INTERACTING RESIDUES
BLIND	**HESPERETIN**	55	-7.75	1.15 µM	8	A	GLN120-ASN123-ASP144-TYR145-PRO271-LEU272-GLN273-ILE280-ARG281-THR282-LYS283
		18	-7.49	1.33 µM	2	C	HIS291-PHE295-LEU308-TYR310-LEU315-GLY321-TYR322-PHE324-ILE325-MET348-LEU353-LEU354-GLYN383
		93	-7.44	1.36 µM	7	D	ASN95-GLY96-GLY97-GLN98-ASP99-VAL126-VAL128-TYR137-LYS244-PHE266-ALA267
		48	-7.67	1.85 µM	4	B	GLN273-PRO275-LEU278-PHE287-ARG288-ASP301-LYS303-ASN366-ILE380-VAL381-THR382
FOCUSED		17	-5.97	21.82 µM	5	C	PHE287-ARG288-THR289-HIS291-CYS300-ARG305-LEU308-LEU315-GLY321-ILE325-HIS337-MET348-PHE351-LEU353-GLN383
		57	-5.84	33.58 µM	36(+17Run89)	B Right side	THR343-GLY344-GLU345-ASN366-ALA367-LEU369-LEU370-TYR371
		92	-5.47	77.24 µM	9	PAS B (left side)	ARG352-TRP360-THR361-TRP362-LEU386-THR387-ASP388

Supplementary Table S5

DOCKING TYPE	LIGAND	RUN	ENERGY (Kcal/mol)	Ki	N° IN CLUSTER	POCKET	INTERACTING RESIDUES
BLIND	**NARINGENIN**	36	-7.94	1.45 µM	2	A	GLN120-LEU122-ASN123-TYR145-LYS283-ILE286-GLN364-SER365-ASN366-THR382-GLN383-ARG384
		24	-7.50	1.70 µM	16	A	GLN120-ASN123-ASP144-TYR145-PRO271-LEU272-GLN273-ILE280
		64	-7.50	1.75 µM	3	PAS A	THR131-GLN171-LEU174-HIS175-PRO180-CYS183-THR184-THR194-GLY195-LEU263
		73	-7.62	2.23 µM	9	B	GLN273-PRO275-LEU278-ARG288-ASP301-LYS303-ILE380-VAL381-THR382
		98	-7.45	3.31 µM	3	B	ASN123-GLN273-ILE286-PHE287-ARG288-ASP301- LYS303-THR382
		62	-7.25	3.55 µM	3	PAS A	THR131-LEU174-HIS175-TRP176-THR194-PRO197-VAL200-PRO260-LEU263
		86	-7.43	3.59 µM	2	PAS A	VAL200-VAL201-CYS202-TYR203-GLN207-TYR245-HIS247-ILE258-PRO260-PRO261
		94	-7.38	3.90 µM	7	A	ASN123-ASP144-TYR145-CYS226-LEU228-ASN230-SER231-GLN273-ILE280
		21	-7.04	4.49 µM	5	C	HIS291-PHE295-LEU308-GLY321-TYR322-PHE324-ILE325-MET348-LEU353-LEU354-GLN383
		63	-6.83	8.79 µM	11	D	ASN95-GLY96-GLY97-GLN98-VAL126-VAL128-TYR137-LYS244-PHE266-ALA267
FOCUSED		40	-5.91	37.83 µM	6 (+2Run97+5Run93)	C	PHE287-ARG288-THR289-HIS291-CYS300-ARG305-LEU308-LEU315-GLY321-ILE325-MET348-LEU353-GLN383
		79	-5.96	38.80 µM	59	B Right side	THR343-ASN366-ALA367-ARG368-LEU369-TYR371
		33	-5.13	161.02 µM	11	PAS B (Left side)	VAL350-ASP329 -ASP388-GLY391

Supplementary Table S6

DOCKING TYPE	LIGAND	RUN	ENERGY (Kcal/mol)	Ki	N° IN CLUSTER	POCKET	INTERACTING RESIDUES
BLIND	CURCUMIN	52	-8.06	645.33 nM	2 (+1Run76)	PAS A	CYS202-TYR203-GLN207-ILE208-PRO210-SER213-PRO214-MET216-LYS242-LEU243-TYR245-HIS247
BLIND	CURCUMIN	50	-7.53	1.01 μM	6	PAS A	THR92-ASN95-ILE208-PRO210-SER213-PRO214-MET216-LYS242-LEU243-LYS244
BLIND	CURCUMIN	72	-7.75	1.25 μM	2	B	ILE280-THR282-LYS283-ILE286-ARG288-ASP301-LYS303-GLN273-LEU278-THR382-ARG384
FOCUSED	CURCUMIN	87	-5.16	127.09 μM	3 (+5Run20+7Run1+5Run10 +19Run4+3Run96+4Run74)	B Right side	GLU345-ASN366-ALA367-ARG368-TYR371-LYS372-ASN373-GLY374
FOCUSED	CURCUMIN	24	-4.76 (LBE=-5.17)	161.18 μM	2	B Left side	GLY347-MET348-ILE349-VAL350-GLN364-SER365-ASN366-ARG384-GLU390-HIS394
FOCUSED	CURCUMIN	16	-4.81 (LBE=-5.15)	167.87 μM	2	Behind B (between PAS A and B)	ARG339-THR343-GLU345-SER346-GLY347-ILE349-VAL350-GLN364-GLU390-GLY391-HIS394

Supplementary Table S7

DOCKING TYPE	LIGAND	RUN	ENERGY (Kcal/mol)	Ki	N° IN CLUSTER	POCKET	INTERACTING RESIDUES
BLIND	QUERCETIN	48	-7.15	2.02 µM	9	A	GLN120-ILE122-ASN123-ASP144-TYR145-LEU235-PRO271-LEU272-GLN273-ILE280
		67	-7.46	3.37 µM	2	A	ASN123-ASP144-TYR145-LEU228-ASN230-GLN273-GLU279-ILE280
		29	-7.21	3.73 µM	6	B	ARG268-GLN273-PRO275-PHE287-ASP301-LYS303-THR382
		87	-6.84	3.92 µM	15	PAS A	GLN207-ILE208-PRO209-SER213-MET216-LYS242-LEU243-LYS244-TYR245
		23	-6.64	4.89 µM	3	A	GLN120-ALA121-ASN123-TYR145-GLN273-LEU278-ASN366-THR382-ARG384
		97	-6.94	5.33 µM	4	Between A and B	ASN123-GLN273-PRO274-PRO275-SER276-LEU278-ASN366-THR382
		99	-6.41	12.68 µM	14	D	ASN95-GLY97-GLN98-VAL126-VAL128-LYS244-PHE266-ALA267
		43	-6.64	13.34 µM	2	C	THR289-HIS291-PHE295-LEU308-TYR310-LEU315-GLY321-PHE324-ILE325-MET348-LEU353-LEU354-GLN383
FOCUSED		7	-6.21	25.27 µM	8	C	HIS291-PHE295-GLY321-ILE325-HIS337-MET340-MET348-LEU353-SER365-ALA367-THR378-VAL381-GLN383
		74	-5.55	39.61 µM	26	B Right side	GLU345-ASN366-ALA367-ARG368-LEU369-LEU370-TYR371-ILE380
		87	-5.17	85.19 µM	16	B Right side	THR343-GLY344-GLU345-ASN366-ALA367-ARG368-LEU369-LEU370-TYR371
		90	-5.11	137.70 µM	19	PAS B (Left side)	ASP329-ILE349-VAL350-TRP362-ASP388-GLY391-THR392-HS394
PARTIALLY FOCUSED		46	-7.41	1.50 µM	15	A	GLN120-LEU122-ASN123-ASP144-TYR145-LEU235-PRO271-GLN273-ILE280
		52	-7.15	4.23 µM	12	B	GLN273-PRO275-PHE287-ARG288-ASP301-LYS303-VAL381-THR382

Supplementary Table S8

DOCKING TYPE	LIGAND	RUN	ENERGY (Kcal/mol)	Ki	N° IN CLUSTER	POCKET	INTERACTING RESIDUES
BLIND	**RESVERATROL**	69	-7.33 (LBE=-7.82)	1.86 μM	11	B	GLN273-PRO275-THR282-PHE287-ARG288-ASP301-LYS303-ILE380-VAL381-THR382 (4H-BONDS)
		64	-6.92	3.61 μM	8	PAS A	THR131-TYR159-GLN171-LEU174-HIS175-TRP176-PRO180-THR184-VAL200-LEU263
		49	-6.90	4.90 μM	2	PAS A	THR131-TYR159-GLN171-LEU174-HIS175-TRP176-PRO180-CYS183-THR184-LEU263
		99	-6.78	7.37 μM	7(+7Run100)	PAS A	CYS202-GLN207-SER213-LEU215-MET216-PRO210-LYS242-LEU243-TYR245-HIS247
		79	-6.71	8.64 μM	11(+11Run28)	A	GLN120-ALA121-LEU122-TYR145-PRO271-GLN273-ILE280-ARG281-THR282-LYS283
		84	-6.66	9.56 μM	3	Between A and B	GLN120-ASN123-ASP144-TYR145-GLN273-LEU278-ASN366-THR382
		62	-6.54	9.66 μM	3 (+3Run)	D	GLY96-GLY97-GLN98-ASP99-VAL126-VAL128-TYR137-GLN240-GLY241-PHE266-ALA267-ILE268
		28	-6.66	9.71 μM	11	A	GLN120-ALA121-ASN123-GLN273-LEU276-ARG281-THR282-LYS283-ILE286-THR382-ARG384
FOCUSED		74	-5.72	41.55 μM	11 (+15Run19+10Run70)	C	PHE287-HIS291-PHE295-LEU308-GLY321-HIS337-PHE351-LEU353-GLN383
		50	-5.51	71.58 μM	53	B Right side	GLY344-GLU345-ASN366-ALA367-ARG368-LEU369-ARG371-ILE380

Supplementary Table S9

DOCKING TYPE	LIGAND	RUN	ENERGY (Kcal/mol)	Ki	N° IN CLUSTER	POCKET	INTERACTING RESIDUES
BLIND	**CYANIDIN-3-GLUCOSIDE**	62	-6.88	1.39 µM	2 (+3Run88+2Run49 +3Run95)	PAS A	SER90-THR92-ASN95-GLN207-LYS242-LEU243-LYS244-TYR245
		35	-5.04 (LBE=-6.47)	18.23 µM	5 (+2Run80)	PAS A	PRO209-PRO210-SER213-MET216-LYS242-LEU243-LYS244
		52	-5.53 (LBE=-5.98)	41.63 µM	2	D	PHE83-GLY96-GLY97-GLN98-ASP99-VAL126-VAL128-PHE136-TYR137-PHE266
		18	-5.7	66.01 µM	1	D (Between PAS A and B)	GLU114-PHE117-ALA121-ARG384-THR387-ASP388
		98	-5.18	160.27 µM	1	D (Between PAS A and B)	LEU122-LEU118-VAL350-TRP362-ARG384-PRO385-LEU386-THR387
FOCUSED		96	-5.15 (LBE=-6.52)	16.51 µM	6	PAS B (Left side)	ASP329-ILE349-VAL350-TRP362-ASP388-GLY391-THR392-HIS394 (5H-BONDS)
		7	-4.00	388.58 µM	4	B Right side	THR343-GLY344-GLU345-ASN366-ALA367-ARG368-LEU369-ILE380

Supplementary Table S10

DOCKING TYPE	LIGAND	RUN	ENERGY (Kcal/mol)	Ki	N° IN CLUSTER	POCKET	INTERACTING RESIDUES
BLIND	**EPIGALLOCATECHIN-3-GALLATE**	57	-8.91	295.29 nM	1 (+2Run39)	PAS A	PHE82-THR92- ASN95-CYS202-GLN207-ILE208-PRO209-SER213-LEU215-MET216-LYS242-LEU243-LYS244-TYR245-HIS247-PHE266
		70	-7.93	1.53 µM	1	D	ARG94-ASN95-GLY96-GLY97-GLN98-ASP99-VAL126-VAL128-TYR137-LYS242-LYS244-PHE266
		72	-7.40	1.66 µM	5	PAS A	ALA86-SER90-GLN207-PRO209-PRO210-MET216-LYS242-LEU243-LYS244-TYR245-HIS247
		33	-6.65	2.77 µM	2	B	ASN123-GLN273-PRO275-SER276-LEU278-THR282-ARG288-LYS303-ILE380-ASN366-THR382
		59	-6.27 (LBE=-7.40)	3.76 µM	5	PAS A	GLN207-PRO209-PRO210-SER213-PRO214-LEU215-MET216-LYS242-LEU243-LYS244-TYR245-HIS247
		80	-5.98 (LBE=-6.98)	7.62 µM	5	D	PHE83-ASN95-GLY96-GLY97-GLN98-ASP99-VAL126-VAL128-TYR137-PHE266
FOCUSED		62	-4.62	47.72 µM	13	B Right side	THR343-GLY344-GLU345-ALA367-LEU369-TYR371-ILE380
		57	-4.59	65.37 µM	8	Behind B (between PAS A and B)	ASP329-VAL350-TRP362-ASP388-GLY391-THR392-HIS394

Supplementary Table S11

DOCKING TYPE	LIGAND	RUN	ENERGY (Kcal/mol)	Ki	N° IN CLUSTER	POCKET	INTERACTING RESIDUES
BLIND	GENISTEIN	59	-7.42	3.65 μM	1(+4Run22)	A<br>(Right side)	ASP144-CYS226-LEU228-ASN230-SER231-SER232-PHE234-LEU235-PRO270-ILE280
		31	-7.08	4.52 μM	2(+2Run89)	PAS A	THR131-LEU174-TYR159-GLN171-HIS175-CYS183-LEU263-
		88	-7.00	5.95 μM	17	A	GLN120-ASN123-ASP144-TYR145-CYS226-LEU235-GLN273-ILE280
		86	-6.82	6.15 μM	28	PAS A	GLN207-ILE208-PRO209-PRO210-SER213-MET216-LYS242-LEU243
		8	-6.84	6.59 μM	9(+5Run17)	Between A and B	LEU122-ASN123-TYR145-GLN273-PRO275-LEU278-THR282-ASN366-THR382
		66	-6.94	7.74 μM	5	PAS A	PHE82-ALA86-SER89-THR92-ASN95-GLY96-LYS244-TYR245-HIS247-PHE266
		90	-6.57	9.85 μM	9	C	PHE287-THR289-HIS291-CYS300-LEU306-TYR310-LEU315-GLY321-TYR322-PHE324-ILE325-MET348-LEU353-GLN383
		24	-6.08	33.25 μM	3	D	GLY96-GLY97-GLN98-VAL126-VAL128-TYR137-GLY241-LYS242- LYS244-PHE266
FOCUSED		15	-6.54	12.29 μM	25	C	PHE287-ARG305-LEU308-TYR310-LEU315-GLY321-ILE325-PHE351-LEU353-GLN383
		49	-5.37	85.29 μM	34<br>(+17Run78)	B<br>Right side	GLY344-ASN366-ALA367-ARG368-LEU369-LEU370-ARG371-ILE380-THR382
		58	-5.24	126.20 μM	11	PAS B<br>(Left side)	ARG352-TRP360-THR361-TRP362-LEU386-ASP388

Supplementary Table S12

DOCKING TYPE	LIGAND	RUN	ENERGY (Kcal/mol)	Ki	N° IN CLUSTER	POCKET	INTERACTING RESIDUES
BLIND	**HYDROXYTYROSOL**	70	-5.24	68.50 µM	8 (+12Run59)	PAS A	PRO210-SER213-LEU215-MET216-LYS242-LEU243-TYR245
		82	-4.60	113.13 µM	5 (+2Run53+3Run81)	B	GLN273-PRO274-PRO275-LEU278-ASN366-ALA367-ILE380-VAL381-THR382
		26	-4.90	188.19 µM	2 (+4Run64+3Run90 +4Run36+5Run46 +3Run29)	A	ASN123-ASP144-TYR145-LEU235-PRO271-GLN273-ILE280
		51	-4.75	204.68 µM	6	PAS A	PHE82-THR92-ASN95-GLY96-LYS244-TYR245-PHE266
		44	-4.79	256.72 µM	2 (+3Run71)	D (Upper side)	ALA79-GLY96-GLY97-GLN98-ASP99-VAL128-PHE136-TYR137-PHE266
FOCUSED		39	-4.15	559.11 µM	15 (+20R.81+ 19R.18)	B	GLY344-GLU345-ASN366-ALA367-ARG368-LEU369-LEU370-ILE380-VAL381-THR382
		95	-3.97	890.60 µM	2 (+7R.88+3R.96 +1R.76)	C	THR289-HIS291-SER320-GLY321-PHE324-ILE325-LEU353-SER365-GLN383

Supplementary Table S13

DOCKING TYPE	LIGAND	RUN	ENERGY (Kcal/mol)	Ki	N° IN CLUSTER	POCKET	INTERACTING RESIDUES
BLIND	**ELLAGIC ACID**	37	-6.77 (LBE=-7.28)	4.60 µM	30 (+5Run43)	Between A and B	<u>GLN120</u>-ASN123-GLN273-LEU278-ILE280-<u>ARG281</u>-THR282-LYS283-ILE286-ASN366-THR382-ARG384
		27	-6.69	7.10 µM	4 (+6Run87+1Run9+3Run65)	PAS A	<u>THR131</u>-TRP176-GLY195-<u>LEU196</u>-PRP197-VAL200-LEU259-PRO260-GLN262
		90	-6.59 (LBE=-6.98)	7.66 µM	7	A	<u>ASN123</u>-ASP144-TYR145-CYS226-LEU228-LEU235-PRO271-LEU272-GLN273-ILE280
		28	-6.82	9.46 µM	5	PAS A	VAL200-VAL201-CYS202-TYR203-<u>GLN207</u>-TYR245-<u>HIS247</u>-PRO260
		46	-6.14 (LBE=-6.56)	15.62 µM	4	B	ASN123-LEU272-<u>GLN273</u>-PRO275-LEU278-THR282-GLU345-ASN366-ALA367-ARG368-ILE380-THR382
		96	-6.23	26.56 µM	12	C	THR289-HIS291-LEU308-TYR310-LEU315-GLY321-PHE324-ILE325-MET348-LEU353-GLN383
FOCUSED		32	-5.05	142.53 µM	36	PAS B (Right side)	THR343-GLY344-<u>GLU345</u>-LEU369-<u>TYR371</u>
		72	-4.89	238.89 µM	32	B	<u>GLU345</u>-ASN366-ALA367-ARG368-ILE380-<u>THR382</u> (3H-BONDS)

Supplementary Table S14

DOCKING TYPE	LIGAND	RUN	ENERGY (Kcal/mol)	Ki	N° IN CLUSTER	POCKET	INTERACTING RESIDUES
BLIND	SULFORAPHANE	23	-5.68	68.88 µM	1 (+3Run79+1Run54)	B	LEU272-GLN273-PRO274-PRO275-LEU278-GLU345-ASN366-ALA367-ILE380-THR382
		66	-4.69	249.79 µM	9 (+6Run56+4Run22+4Run.5)	C	HIS291-PHE295-GLY321-TYR322-ILE325-HIS337-MET348-SER365-SER366
		97	-4.25	278.13 µM	4	PAS A	LEU54-PRO55-PHE56-LEU72-SER75-TYR76-GLN98-ASN100-TYR137
		60	-4.61	286.49 µM	2	PAS A	THR131-TYR159-GLN171-LEU174-HIS175-TRP176-LEU263
		11	-4.42	290.63 µM	4	A	ASN123-TYR145-LEU228-ASN230-LEU235-PRO271-GLN273
		21	-4.40	401.96 µM	7	A (Upper side)	LEU119-GLN120-LEU122-ASN123-THR141-TYR145-LYS283-ARG384 (2H-BONDS)
FOCUSED		4	-3.99 (LBE=-4.40)	593.22 µM	26 (+4Run84+9Run34 +5Run6+4Run49)	C	THR289-HIS291-GLY304-ARG305-LEU308-TYR310-LEU315-SER320-PHE324-LEU353
		26	-3.97 (LBE=-4.27)	744.21 µM	5	DOPO B	GLY344-GLU345-ASN366-ALA367-ARG368-ILE380-VAL381-THR382
